# Mechanisms of regulation of glycolipid metabolism by natural compounds in plants: effects on short-chain fatty acids

**DOI:** 10.1186/s12986-024-00829-5

**Published:** 2024-07-18

**Authors:** Jiarui Li, Jinyue Zhao, Chuanxi Tian, Lishuo Dong, Zezheng Kang, Jingshuo Wang, Shuang Zhao, Min Li, Xiaolin Tong

**Affiliations:** 1 College of Traditional Chinese Medicine, Changchun University of Chinese Medicine, Changchun, China; 2 Beijing University of Chinese Medicine, Beijing, China; 3 The Affiliated Hospital, Changchun University of Chinese Medicine, Changchun University of Chinese Medicine, Changchun, China; 4 Research Laboratory of Molecular Biology, Guang’anmen Hospital of China Academy of Chinese Medical Sciences, Beijing, China; 5 Guang’anmen Hospital, Academician of Chinese Academy of Sciences, China Academy of Traditional Chinese Medical Sciences, Beijing, China

**Keywords:** Natural compounds, Short-chain fatty acids, Glucose metabolism, Lipid metabolism

## Abstract

**Background:**

Natural compounds can positively impact health, and various studies suggest that they regulate glucose‒lipid metabolism by influencing short-chain fatty acids (SCFAs). This metabolism is key to maintaining energy balance and normal physiological functions in the body. This review explores how SCFAs regulate glucose and lipid metabolism and the natural compounds that can modulate these processes through SCFAs. This provides a healthier approach to treating glucose and lipid metabolism disorders in the future.

**Methods:**

This article reviews relevant literature on SCFAs and glycolipid metabolism from PubMed and the Web of Science Core Collection (WoSCC). It also highlights a range of natural compounds, including polysaccharides, anthocyanins, quercetins, resveratrols, carotenoids, and betaines, that can regulate glycolipid metabolism through modulation of the SCFA pathway.

**Results:**

Natural compounds enrich SCFA-producing bacteria, inhibit harmful bacteria, and regulate operational taxonomic unit (OTU) abundance and the intestinal transport rate in the gut microbiota to affect SCFA content in the intestine. However, most studies have been conducted in animals, lack clinical trials, and involve fewer natural compounds that target SCFAs. More research is needed to support the conclusions and to develop healthier interventions.

**Conclusions:**

SCFAs are crucial for human health and are produced mainly by the gut microbiota via dietary fiber fermentation. Eating foods rich in natural compounds, including fruits, vegetables, tea, and coarse fiber foods, can hinder harmful intestinal bacterial growth and promote beneficial bacterial proliferation, thus increasing SCFA levels and regulating glucose and lipid metabolism. By investigating how these compounds impact glycolipid metabolism via the SCFA pathway, novel insights and directions for treating glucolipid metabolism disorders can be provided.

## Introduction

Glucose metabolism and lipid metabolism are important processes in the maintenance of energy homeostasis and normal physiological functions and play crucial roles in maintaining intracellular homeostasis and energy balance [1]. The molecular mechanism of glucose metabolism involves multiple steps such as glucose uptake, glycogen synthesis, glycogenolysis, glycolysis, and the tricarboxylic acid cycle [2]. During fasting, glycogenolysis is the main source of glucose released into the bloodstream [3]. Disorders of glucose metabolism are characterized mainly by hyperglycemia, dyslipidemia, fatty liver, and atherosclerosis [4]. Lipids, including triglycerides, phospholipids and steroids, are important components of the body [5]. The regulation of lipid metabolism, such as lipid uptake, synthesis and hydrolysis, is essential for the maintenance of cellular homeostasis [6]. Dysregulation of lipid metabolism in the human body can cause a variety of diseases, such as hyperlipidemia [7], osteoporosis [8], atherosclerosis [8], obesity and diabetes [9].

Natural compounds are widely distributed in plants and their derived materials [10]. Natural compounds are secondary metabolites of plants [11], and they include nutrients essential for health, such as proteins, carbohydrates, vitamins, minerals and other chemicals, such as phenolic acids, flavonoids and other phenolic substances [12]. The ability of natural compounds to prevent chronic diseases by preventing oxidative stress and inflammation, inducing autophagy, and interacting with the gut microbiota, among other signaling pathways, and their nutritional effects have been widely studied [13]. Natural compounds have been shown to regulate the body’s metabolism and reduce the risk of chronic diseases such as type 2 diabetes, atherosclerosis and cancer [14]. Recent studies have indicated that many natural compounds affect the production of short-chain fatty acids (SCFAs), thereby ameliorating disorders of glucolipid metabolism [15, 16].

SCFAs are produced by anaerobic fermentation of dietary fiber and resistant starch by the microbiota in the gut [17]. SCFAs are a type of saturated fatty acid, among which acetic, propionic and butyric acids are the most abundant SCFAs in the human body and can maintain the integrity of the intestinal barrier and influence the production of gastrointestinal mucus [18]. Studies in recent years have shown that SCFAs play important roles in human energy metabolism and energy supply [19]. As a microbial metabolite, they can maintain host health through mechanisms related to the regulation of gut barrier function, microbial activity, and glucose homeostasis [20]. Short-chain fatty acids can prevent and manage diabetes by increasing insulin sensitivity, improving glucose homeostasis and inhibiting hepatic gluconeogenesis [21]. SCFAs also inhibit the production of fat [22]. Acetate reduces lipid accumulation, inhibits the lipolysis of white adipose tissue and induces browning of white adipose tissue, which reduces body fat by increasing thermogenesis [23]. Increasing SCFA levels may be an effective therapeutic modality against dysglycolipidemia [24]. SCFAs may regulate glucose homeostasis by decreasing glucose production [25], increasing glucose uptake and glycogen synthesis in the liver [26], increasing pancreatic β-cell mass and regulating insulin secretion [27]. In regulating lipid metabolism, short-chain fatty acids may improve lipid metabolism by decreasing inflammation in adipose tissue [28], increasing the lipid buffering capacity of adipose tissue, and enhancing fatty acid oxidation and mitochondrial function in the liver and skeletal muscle [24].

In this paper, we comprehensively review the relevant roles of SCFAs in glycolipid metabolism; introduce potential natural compounds that may ameliorate the dysregulation of glycolipid metabolism by modulating the SCFA pathway, such as polysaccharides, anthocyanins, quercetin, resveratrol, carotenoids, and betaines; and briefly discuss the ways in which each of these compounds influences glycolipid metabolism through the SCFA pathway, with the goal of providing new ideas for the treatment of glycolipid metabolism dysregulation. We briefly discuss how each compound affects glycolipid metabolism through SCFAs to provide new ideas for the treatment of dysglycolipidemia.

## Generation of SCFAs

SCFAs are produced by the fermentation of indigestible dietary components, including complex carbohydrates [29] and dietary fiber, by the gut microbiota. The colon is the primary site of SCFA production within the human body, as it harbors the highest density of the gut microbiota [30]. While SCFAs are derived primarily from the fermentation of microbially accessible carbohydrates (MACs), they can also be byproducts of bacterial amino acid metabolism. The relative contribution of amino acid metabolism to overall SCFA production is not well understood, but the total intake of protein and fiber are considered influential factors [31]. Owing to the physiological pH range of the colonic lumen, which typically falls between 5.6 and 6.6, the majority of SCFAs exist in their anionic form, rendering simple diffusion challenging [32]. After production, SCFAs are primarily transported from the colonic lumen into the colonic cells through passive diffusion and/or carrier-mediated transport [33]. SCFA concentrations vary along the length of the colon, with the highest levels observed in the cecum and proximal colon and a decline in concentration toward the distal colon. This gradient is likely due to the increased absorption of SCFAs mediated by the sodium-coupled monocarboxylate transporter SLC5A8 and the low-affinity H+-coupled monocarboxylate transporter SLC16A1 [34]. It may also be due to the greater availability of carbohydrates and water in the proximal portion of the colon than in the distal portion. The total amount of SCFAs in the proximal colon is estimated to be 70–140 mM, whereas the total amount of SCFAs in the distal colon decreases to 20–70 mM [35, 36].

SCFAs are carboxylic acids containing 1–6 carbon atoms, including acetic, propionic, butyric, valeric, and caproic acids, of which acetic (C2), propionic (C3), and butyric (C4) acids are the most abundant and are produced by anaerobic fermentation of dietary fiber (DF) in the intestines [35, 37]. The molar ratio of C2, C3 and C4 is approximately 60:20:20 [38]. Acetate is the major anion in human intestinal contents, accounting for more than 50-60% of short-chain fatty acids, followed by propionate and butyrate in roughly equal amounts, with small amounts of the branched-chain fatty acids isobutyric and isovaleric acids, as well as small amounts of lactic acid and succinic acid [35, 39].

The acetic acid production pathway is widely distributed in the bacterial community, whereas the propionic, butyric, and lactic acid production pathways are more conserved and substrate specific [40]. The main dietary sources of acetate are acetate-containing foods such as pickles, cheese and other dairy products; processed meats; bread; wine; beer; and bacterial breakdown of dietary fibers such as resistant starch, indigestible oligosaccharides and other plant polysaccharides. Although fructose is mainly absorbed from the small intestine, unabsorbed fructose reaches the colon, where it can be converted by the microbiota to acetic acid. Bacteria such as *A. muciniphila* and *Bacteroides spp*. produce acetic acid by digesting food fibers in the colon. The gut bacteria that produce acetic acid also include *Bifidobacterium spp.*, *Akkermansia muciniphila*,* Pravotella spp.* and *Ruminococcus spp.* After dietary fiber reaches the intestinal tract, the intestinal microbiota breaks down the dietary fiber through two metabolic pathways, glycolytic fermentation or acetogenesis, to produce acetate. Most of these species of gut bacteria produce acetic acid by fermenting pyruvic acid through acetyl-CoA. In addition, acetic acid-producing bacteria can also produce acetic acid from CO2 and H2 via the Wood–Ljungdahl pathway. Acetate can also be derived from the metabolism of the oxidative breakdown of alcohol in the liver. Acetic acid may also be produced from microbial fermentation of residual peptides and fats [35, 41–45].

Propionic acid is produced mainly by *Bifidobacterium spp.* and mucin-degrading bacteria such as *Akkermansia muciniphila* [37]. There are three pathways for propionate formation in human gut bacteria: the succinate pathway, the acrylate pathway, and the propylene glycol pathway [46]. Among them, the phylum *Bacteroidetes* utilizes the succinate pathway to produce propionic acid via methyl propionyl coenzyme A. Negative bacteria in the *Firmicutes phylum* also utilize the succinate pathway to produce propionic acid. In addition, propionic acid can be formed from organic acids, such as the succinic acid in organic acids. In addition, *Megasphaera elsdenii* produces butyric acid when it is grown on glucose but produces propionic acid when it is grown on lactate. Some bacteria can also produce 1,2-propanediol from oligosaccharides, dihydroalcoholic phosphate or lactic acid, which is then further metabolized to propionic acid. Propionate is generated from fucose via propylene glycol in *Roseburia inulinivorans* from the human gut [47].

Butyric acid is produced by intestinal bacteria of the genera *Faecalibacterium prausnitzii*, *Eubacterium rectale* and *Roseburia spp.* [48] and by *Ruminococcaceae*, *Lachnospiraceae*, *Anaerobutyricum hallii* and *Anaerostipes spp.* [49]. Butyric acid is the preferred energy source for colonic epithelial cells and plays an important role in their metabolism and normal development [50]. Butyric acid is derived from carbohydrates through glycolysis and produced from the combination of two molecules of acetyl coenzyme A to form acetoacetyl coenzyme A, which is then progressively reduced to butyryl coenzyme A. There are two different pathways for the formation of butyric acid from butyryl coenzyme A, either by butyryl coenzyme A, acetate coenzyme transferase or phosphotransbutyrylase and butyric acid kinase [51]. Resistant starch (RS) is the main source of butyrate [52]. A study by Venkataraman et al. [53]. reported that dietary supplementation with resistant starch increases fecal butyrate concentrations. A study by Louis et al. [54]. revealed that although *Faecalibacterium prausnitzii* accounts for approximately 10% of human fecal bacteria, it accounted for only 4% of the butyl coenzyme A transferase sequences identified in their study, suggesting that only a few strains of *Fraecalibacterium prausnitzii* have the butyryl coenzyme A and acetate coenzyme transferase genes and that the majority of strains are not butyrate producers.

## SCFAs and human glucose and lipid metabolism

Short-chain fatty acids constitute approximately 10% of human caloric needs [35] and play important roles in the regulation of glucose metabolism and lipid metabolism [19]. Glycometabolism refers mainly to the way blood glucose is metabolized, which is derived from intestinal absorption, hepatic glycogenolysis, and gluconeogenesis [55]. Under fed conditions, carbohydrates in food are digested and processed by various glucosidases in the digestive tract, and the resulting monosaccharides are transported to various tissues as the main fuel for ATP production [56]. The liver plays a major role in controlling glucose homeostasis by controlling various pathways of glucose metabolism, such as gluconeogenesis, glycogenolysis, glycolysis, and gluconeogenesis, with glycolysis being essential for most of the cells where glucose catabolism for energy production is crucial [57]. Glucose is phosphorylated by hexose kinase to form glucose 6-phosphate, which enters the glycolytic pathway and undergoes a series of enzyme-catalyzed reactions to produce pyruvate, along with ATP for energy or glycogen storage [19]. Whereas lipid metabolism refers mainly to the way in which lipids are metabolized, lipids are stored mainly in adipose and other tissues or in other nonadipose tissues; excessive accumulation of triacylglycerol (TAG) and cholesteryl esters (CE) leads to abnormalities in lipid metabolism [55], and inactivation of the synthesis of TAG, a major energy substrate stored in adipose tissues, leads to temporal and spatial variations in fat absorption and a reduction in postprandial triglyceridemia, postprandial changes in gut hormone levels, and resistance to diet-induced obesity in rodents [58]. In contrast, elevated levels of cholesterol in the circulation are among the major risk factors for atherosclerosis [59].

SCFAs are able to regulate glucose and lipid metabolism by acting on the G protein-coupled receptors GPR43 and GPR41 in the terminal ileum and colon [60], with propionate being the most potent agonist of GPR41 and GPR43. Acetic acid is more selective for GPR43, whereas butyric and isobutyric acids are more active for GPR41 [61]. Andrew J. Brown et al. [62]. reported that valerate (C5) activated GPR41 more effectively than did acetic acid, but acetic acid activated GPR43 more effectively than did valeric acid. GPR43 is also known as free fatty acid receptor 2 (FFAR2). Ikuo Kimura et al. [63]. reported that short-chain fatty acid-mediated activation of GPR43 could inhibit lipid accumulation and ameliorate obesity. GPR41 is also known as free fatty acid receptor 3 (FFAR3). Ikuo by Kimura et al. [64] reported that propionate was able to maintain metabolic homeostasis and body energy expenditure by directly modulating sympathetic nervous system (SNS) activity through GPR41 at the sympathetic ganglion level.

### Glucoregulatory functions of SCFAs in different metabolic tissues

In the liver, Shoji Sakakibara et al. [65]. reported that sodium acetate directly activates AMPK by neutralizing acetic acid (AcOH), which in turn reduces the expression of genes such as glucose-6-phosphatase (G-6-pase) and sterol regulatory element-binding protein-1 (SREBP1) in rat liver cells. Research by Huating Li et al. [66] indicated that butyrate can increase fibroblast growth factor 21 (FGF21) levels in the liver, stimulating the oxidation of long-chain fatty acids and decreasing glucose levels. Short-chain fatty acids (SCFAs) are capable of increasing the mRNA expression of glucose transporter 2 (GLUT-2) and glycogen synthase 2 (GYS2) in the liver, thereby reducing hepatic glycolysis and gluconeogenesis while increasing glycogen synthesis [17, 67].

In adipose tissue, SCFAs can promote the release of adiponectin by increasing the expression of GPR41 and GPR43 [68, 69], and adiponectin can increase glucose metabolism during glycogen breakdown in the liver, skeletal muscle, and brown adipose tissue (BAT) [70]. A study by Ikuo Kimura et al. [71] revealed that GPR43 activation mediated by short-chain fatty acids could inhibit insulin signaling in adipocytes, whereas acetate could suppress glucose uptake in adipocytes and promote glucose metabolism in other tissues.

Skeletal muscle is considered the largest organ in the body and is responsible for approximately 80% of insulin-stimulated glucose uptake [72]. An increase in SCFAs can activate AMP-activated protein kinase (AMPK), leading to improved insulin sensitivity and increased glucose metabolism in skeletal muscle [73]. This phenomenon is likely attributed to the ability of AMPK to regulate the expression of GLUT4 [74], which is the predominant glucose transporter in skeletal muscle cells [19]. Glucose is transported into muscle cells through the GLUT4 transporter, thereby influencing glucose uptake [74]. Furthermore, a study by T Fushimi et al. [75] indicated that in skeletal muscle, acetate may inhibit glycolysis by suppressing the activity of phosphofructokinase-1 (PFK-1).

In the gut, SCFAs act as promoters for two crucial gut hormones, glucagon-like peptide-1 (GLP-1) and peptide YY (PYY) [17, 76]. SCFAs can increase the secretion of plasma GLP-1 by upregulating the expression of GPR41, GPR43, PC1/3, and GCG. Subsequently, GLP-1 induces insulin secretion and inhibits glucagon secretion, effectively regulating and controlling blood glucose metabolism [77, 78]. SCFAs increase the expression of PYY in enteroendocrine cells within the gut through two distinct pathways. Acetate, propionate, and butyrate stimulate GPR43, resulting in a slight increase in PYY mRNA levels. Additionally, propionate and butyrate induce a significant increase in PYY mRNA levels by inhibiting histone deacetylases (HDACs). PYY plays a critical role in regulating food intake and insulin secretion [79].

SCFAs can also increase the sense of satiety through the gut–brain axis. Acetate can inactivate AMP-activated protein kinase (AMPK) in the hypothalamus, leading to increased activity of acetyl-CoA carboxylase (ACC). This process, in turn, stimulates the expression of proopiomelanocortin (POMC) and predominantly GABAergic neurotransmission in the hypothalamus while reducing the expression of neuropeptide Y (NPY) and agouti-related peptide (AgRP). This cascade ultimately decreases appetite and food intake, preventing weight gain and reducing the risk of type 2 diabetes mellitus (T2DM) [17, 80]. SCFAs can also control glucose and energy homeostasis through the gut–brain neural axis. Propionate activates the fatty acid receptor FFAR3 (GPR41), leading to increased c-Fos expression (a well-recognized marker of neuronal activation) and the activation of intestinal gluconeogenesis (IGN) gene expression through neural pathways, such as the dorsal vagal complex (DVC), the C1 segment of the spinal cord, the parabrachial nucleus (PBN), and the hypothalamus. Butyrate, conversely, increases the expression of cyclic adenosine monophosphate (cAMP), which induces upregulation of the glucose-6-phosphatase catalytic subunit (G6PC) and phosphoenolpyruvate carboxykinase 1 (PCK1) genes, directly activating IGN gene expression in intestinal cells [81]. Acetate can also provide fuel for the tricarboxylic acid (TCA) cycle in central nervous system (CNS) microglia. Studies have shown that germ-free (GF) mice have significantly lower acetate levels, possibly because microglia utilize acetate to produce oxaloacetic acid (OAA), activating ATP-citrate lyase (ACL)-mediated conversion of citrate to acetyl-coenzyme A (Ac-CoA) as a carbon donor in the TCA cycle, the production of citrate and the generation of ATP [82].

Glycolysis is a primary means by which normal cells obtain energy under aerobic conditions. In the presence of oxygen, normal cells generate energy through mitochondrial oxidative phosphorylation (OXPHOS). However, when oxygen is limited, cells rely on glycolysis to produce ATP. In contrast, cancer cells undergo metabolic reprogramming to prioritize glycolysis for energy production, even in the presence of sufficient oxygen [83, 84]. Histone deacetylases (HDACs) are evolutionarily conserved enzymes that remove acetyl modifications from histones, playing crucial roles in epigenetic gene silencing [85]. HDAC inhibitors can suppress c-Myc protein levels and increase the expression of proliferator-activated receptor γ coactivator 1α (PGC1α) and peroxisome proliferator-activated receptor δ (PPARδ), driving oxidative energy metabolism. This process leads to an increase in fatty acid oxidation (FAO) and oxidative phosphorylation (OXPHOS) while weakening glycolysis and reducing ATP levels [86]. As HDAC enzyme inhibitors, short-chain fatty acids (SCFAs) can epigenetically regulate cellular metabolism [34]. Valerate and butyrate can increase the activity of mTOR (mechanistic target of rapamycin) and inhibit class I HDAC enzymes, leading to increased expression of CD25, interferon gamma (IFN-γ), interleukin-2 (IL-2), and tumor necrosis factor alpha (TNF-α) in cytotoxic T lymphocytes (CTLs) through the glycolytic pathway, thereby enhancing cellular antitumor immunity [87]. Additionally, mTORC1-driven glutamine uptake can suppress the expression of glycolytic genes, such as Slc2a1 (glucose transporter 1), the hexokinase isoforms Hk2 and Hk3, and glucose metabolism in cancer cells [88].

**Fig. 1:**
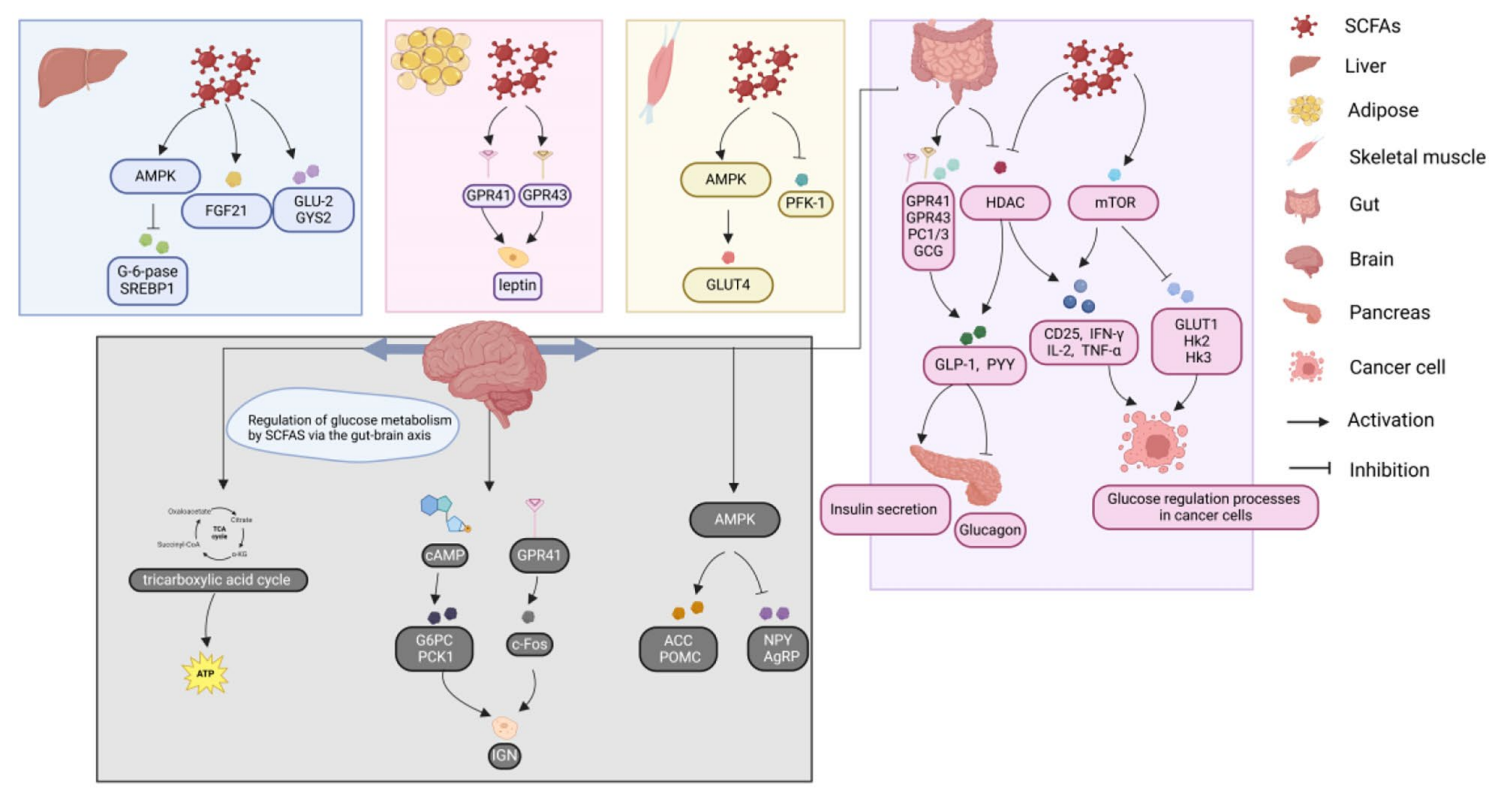
Glucose metabolism is affected by SCFAs

In summary, SCFAs in different metabolic tissues are able to regulate glucose metabolism by decreasing glycolysis and gluconeogenesis, increasing insulin secretion, and increasing glycogen synthesis (Fig. 1).

SCFAs have systemic effects on glucose metabolism to varying degrees in different tissues. The major metabolic pathways include the liver, adipocytes, skeletal muscle, intestine, pancreas and gut-brain axis, impacting the human liver through glucose transport, FGF21 and AMPK and adipocytes through GPR41 and GPR43; skeletal muscle through AMPK and PFK-1; and the human intestine via GPR41, GPR43, PC1/3 and GCG to subsequently affect the brain via the brain‒gut axis.

### Lipid regulatory functions of SCFAs in different metabolic tissues

In the context of the liver, a study by Hua Zhou et al. [67] demonstrated that the exogenous provision of short-chain fatty acids (SCFAs) can increase the protein levels of GPR43 and reduce the protein levels of ACC within hepatic tissue. Concurrently, SCFA supplementation was observed to upregulate the expression of AMPK, collectively contributing to the improvement of lipid metabolism. Furthermore, there is evidence suggesting that acetate can induce the phosphorylation of AMPKα, which in turn leads to the induction of PPARα expression in hepatocytes. This cascade of events ultimately suppresses the expression of SREBP-1c and ChREBP, thereby inhibiting the mRNA expression of lipogenic genes and reducing fatty acid synthesis in the liver [89, 90].

In the context of adipose tissue, a study by Johan W E Jocken et al. [91] revealed that acetate could attenuate the phosphorylation of hormone-sensitive triglyceride lipase (HSL) in adipocytes, thereby modulating lipid metabolism in human adipose tissue. Furthermore, research conducted by Zhanguo Gao et al. [92] revealed that butyrate could increase the expression of two thermogenesis-related genes, PGC-1α and UCP-1, in brown adipose tissue (BAT). Additionally, a study by Gijs den Besten et al. [93] demonstrated that supplementation with short-chain fatty acids (SCFAs) could reduce the expression of peroxisome proliferator-activated receptor γ (PPARγ) and increase the expression of UCP-2 in adipocytes, ultimately stimulating the oxidative metabolism of adipose tissue through activation of AMPK. Importantly, existing evidence [94] suggests that mitochondrial uncoupling in adipocytes plays a crucial role in the regulation of lipid metabolism and obesity, with mitochondrial uncoupling protein 1 (UCP-1) being the most significant marker of BAT. Upregulation of UCP-1 expression can promote lipid consumption and heat generation [95]. Furthermore, mitochondrial uncoupling protein 2 (UCP-2) is expressed in various tissues, including the spleen, kidneys, immune system, pancreas, and central nervous system, and it can also contribute to the promotion of lipid metabolism [96].

UCP3 is the predominant UCP isoform in skeletal muscle [97]. Mitochondrial dysfunction in skeletal muscle may be a contributing factor to impaired lipid oxidation, which is associated with decreased expression of the UCP3 protein in muscle [98]. A study by Jian Hong et al. [99] revealed that butyrate could increase the content of muscle by increasing the expression of UCP-2, UCP-3, and fatty acid oxidation enzymes such as recombinant carnitine palmitoyltransferase 1b (CPT1-b) and peroxisome proliferator-activated receptor gamma coactivator-1α (PGC1-α) in skeletal muscle. Furthermore, SCFAs can increase the production of muscle in children by increasing the expression of interleukin-15 (IL-15), a myokine associated with muscle growth [100].

In the intestinal tract, SCFAs act on the GPR41 receptor to increase the release of the satiety hormone PYY in the colon, which has been shown to increase fasting lipid oxidation [101, 102]. Interestingly, a study by Zhuang Li et al. [103]] demonstrated that the ingestion of butyrate can stimulate the secretion of GLP-1 in the intestine, thereby activating GLP-1 receptor signaling in the vagus nerve. This process, in turn, reduces the activity of the orexigenic neuropeptide Y (NPY) in the hypothalamus and the neurons in the nucleus of the solitary tract (NTS) and the dorsal vagal complex (DVC) through the gut–brain axis, leading to decreased food intake and increased satiety, ultimately preventing obesity and fat accumulation. Additionally, Yibing Zhou et al. [104] reported that valerate can increase the concentration of GPR43 in the colon, which in turn reduces the expression of the NLRP3 inflammasome, TNF-α, and IL-6, thereby decreasing lipid deposition.

**Fig. 2:**
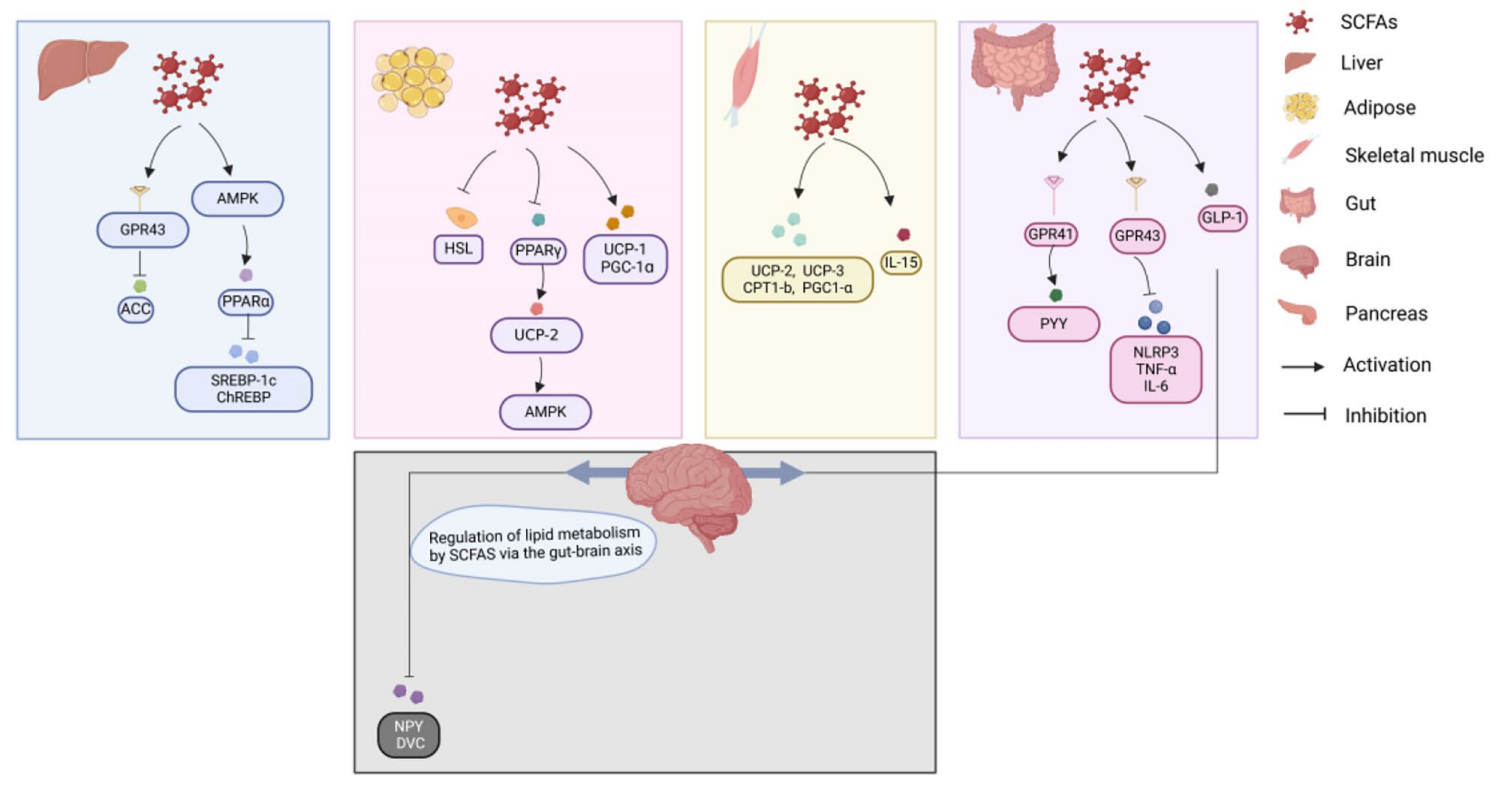
Lipid metabolism is influenced by SCFAs

In conclusion, SCFAs in different metabolic tissues are able to regulate lipid metabolism by promoting lipid oxidation, reducing lipid synthesis, decreasing lipid deposition and increasing thermogenesis (Fig. 2).

SCFAs have different systemic effects on lipid metabolism at different levels. The main metabolic pathways include the liver, adipocytes, skeletal muscle, intestine, pancreas and gut-brain axis, impacting the human liver via GPR43 and AMPK. Additionally, adipocytes are affected via HSL, PPARγ, UCP-1, UCP-2, PGC-1ɑ and AMPK; skeletal muscle is affected via UCP-2, UCP-3, CPT1-b, PGC1-α and IL-15; and the gut is affected via GPR41, GPR43, and GLP-1 to subsequently impact the brain via the brain‒gut axis.

## Natural compounds that regulate glucolipid metabolism

Compounds from various plants are widely used as regulators of human glucose and lipid metabolism and play important roles in the treatment of diabetes, hyperglycemia, obesity, and other diseases [105]. Moreover, natural compounds are widely used in the development of new drugs and play key therapeutic roles in the fields of cancer, infectious diseases, cardiovascular diseases, and multiple sclerosis [106]. In this section, we assess the role of several phytochemicals in regulating human glycolipid metabolism through SCFAs. These phytochemicals include polysaccharides, anthocyanins, quercetin, resveratrol, carotenoids, and betaine. Their basic sources, roles in different metabolic tissues and effects on SCFAs are shown in Table 1.

**Table 1:** Basic sources of six phytochemicals, their roles in different metabolic tissues and their effects on SCFAs

Compound Name	Dietary Source	Role in different metabolic organizations	Effects on SCFAs
Polysaccharides	Seeds	Gut: Altering the concentration of the gut microbiota [110–117]. Downregulation of the expression of TNF-α, and upregulation of the expression of MUC-2 and GPRs [112]. Upregulation of the expression of GLP-1 and PYY [116]. Downregulation of the expression of IL-1β, IL-6, and TNF-α [117].	Upregulation of the concentration of *D. vulgaris*,* llobaculum*,* Lactococcus*,* Muribaculaceae_unclassified*,* Bifidobacterium*,* Alistipes*, *Rikenellaceae_RC9_gut_group*,* Bacteroides Ruminococcus_bromii*,* Anaerotruncus_colihominis*,* Clostridium_methylpentosum*,* Alistipes* and *Odoribacter*. Downregulation of the concentration of *Proteobacteria*,* Lactobacillus*, *Firmicutes_unclassified*,* Dubosiella Bilophila*, and *Streptococcus*.
	Stem and leaf tissues		
	Herbal plants		
	Animal body fluids		
	Cell walls		
Anthocyanins	Vegetative vesicle	Gut: Altering the concentration of the gut microbiota [123–126, 128]. Upregulation of the expression of FFAR2, FFAR3, and TJ [123]. Upregulation of the expression of TJs and NF-κB pathway protein [127].	Upregulation of the concentration of *Ruminococcaceae*,* Akkermansia*,* Bacteroide*,* Odoribacter*,* Roseburia*,* Faecalibaculum*,* Parabacteroides*,* Ruminococcus*,* Intestinimonas*, and *Clostridium_XVIII*. Reduction of the *Firmicutes/Bacteroide* ratio.
Quercetin	Tea, buckwheat	Gut: Altering the concentration of the gut microbiota [133, 134]. Downregulation of the expression of TRPV1, AQP3, and iNOS. Upregulation of the expression of GDNF and c-Kit [133].	Upregulation of the concentration of *Coprococcus*,* Ruminiclostridium* and *Roseburia*. Downregulation of the concentration of *Enterococcus* and *Enterobacter*.
	Cranberries, apples		
	Lettuce, radish leaves, cilantro and onions		
Resveratrol	Grapes and wine	Gut: Altering the concentration of the gut microbiota [142–145]. Downregulation of the expression of IL-6 and IL-1β [143]. Liver: Upregulation of the expression of Nrf2, SOD, and CAT [142].	Upregulation of the concentration of *S24-7*,* Adlercreutzia*,* Faecalibaculum*, and *Lactobacillus*. Downregulation of the concentration of *Allobaculum*,* Blautia*,* Lactobacillaceae*, and *Prevotella.*
	Peanuts, soybeans and berries		
Carotenoids	Vegetables and fruits	Gut: Altering the concentration of the gut microbiota [154]. Liver: Downregulation of the expression of NLRP3, Pro-Caspase-1, Caspase-1, and NF-κB [154].	Upregulation of the concentration of *Allobaculum.* Downregulation of the concentration of *Lachnospiraceae_NK4A136_group*,* Desulfovibrio*, and *Alistipes.*
Betaine	Beta vulgaris	Gut: Altering the concentration of the gut microbiota [159, 160]. Liver: Upregulation of the hepatic lipid oxidation genes PPARα and CPT1α and the hepatic lipid transporter gene FATP2 expression [160]. Adipose: Downregulation of the expression of the adipogenic genes Fas and ACC [160].	Upregulation of the concentration of *A. muciniphila*,* Ruminococcus*,* Oscillospira*,* Lactobacillus*, and *Lactobacillus paracasei*. Downregulation of the concentration of *Aspergillus*,* Desulfovibrio*, and *Ruminococcus.*
	Bran, Wheat Germ, Spinach		

### Polysaccharides

Polysaccharides are naturally occurring macromolecular polymers that usually consist of more than 10 monosaccharides through linear glycosidic or branched chains [107]. Polysaccharides are found in almost all living things in nature, including seeds, stem and leaf tissues, herbal plants, animal body fluids and cell walls [108]. Polysaccharides can lower blood glucose and lipids by repairing pancreatic islet cells, improving insulin resistance, regulating the intestinal flora, enhancing antioxidant capacity, and regulating the activity of key enzymes in glucose and lipid metabolism [109]. A study by Ying Hong et al. [110]. revealed that astragalus polysaccharide was able to increase the content of *D. vulgaris* strains in the midgut of mice fed a high-fat diet, whereas *D. vulgaris* strains were able to significantly increase the content of acetic acid, regulate hepatic lipid metabolism, and effectively attenuate hepatic steatosis in mice. A study by Doudou Li et al. [111]. revealed that acetic acid levels were elevated in high-fat chow-fed mice after 12 weeks of LBP supplementation, and the administration of moderate and high doses of LBP significantly lowered blood glucose and increased fasting serum insulin levels. These findings suggested that acetic acid may bind to receptors on intestinal neurons and regulate duodenal hypercontractility, thereby improving glucose homeostasis. A study by Xinyi Tian et al. [112]. revealed that polysaccharides increase the relative abundance of *Allobaculum* and *Lactococcus*, decrease the relative abundance of *Proteobacteria*, and increase the level of SCFAs and the expression of related G-protein-coupled receptors in the intestinal bacterial community of mice fed a high-sugar and high-fat diet. A study by Jinli Xie et al. [113]. revealed that *Ganoderma lucidum* polysaccharides significantly increased the levels of propionic acid and butyric acid in the small intestine and cecum of rats, thereby enhancing intestinal immunity and reducing inflammatory reactions. A study by Ying Lan et al. [114]. revealed that Seabuckthorn polysaccharides increase the proportions of *Muribaculaceae_unclassified*, *Bifidobacterium*, *Alistipes*, and *Bacteroides* in the intestines of high-fat diet-induced obese mice; and decrease the proportions of intestinal *Lactobacillus*, *Firmicutes_unclassified*, *Dubosiella Bilophila*, and *Streptococcus*, which are also able to increase the content of SCFAs in feces and regulate hepatic lipid metabolism by modulating changes in the gut microbiome and the SCFA content. A study by Liman Luo et al. [115]. revealed that inulin-type fructans could increase fecal and serum acetate concentrations, reducing mitochondrial dysfunction and toxic glucose metabolite levels. A study by Ye Yao et al. [116]. revealed that srychnine polysaccharides were able to increase the production of SCFAs by SCFA-producing bacteria, such as *Ruminococcus bromii*, *Anaerotruncus_colihominis*, and *Clostridium_methylpentosum*, and upregulate GLP-1 and PYY to improve glucose metabolism in rats. A study by Ciliang Guo et al. [117]. revealed that hawthorn polysaccharides increased the proportions of *Alistipes* and *Odoribacter* in the intestinal tract microbiota, increased the contents of acetic acid and propionic acid, and inhibited the expression of inflammatory cytokines such as interleukin-1β (IL-1β), interleukin-6 (IL-6) and tumor necrosis factor alpha (TNF-α).

### Polyphenols

Polyphenols are among the most abundant and widely distributed natural products in the plant kingdom [118]. They are phytochemicals that are synthesized from plants with one aromatic ring and one hydroxyl group and are found in large quantities in various natural plants, including fruits and vegetables. Polyphenols promote health and prevent various types of chronic diseases. They have the ability to regulate signaling pathways and exert antioxidant activities, which can regulate processes such as oxidative stress, inflammation and apoptosis [119]. According to their different chemical structures, polyphenols can be categorized into phenolic acids, flavonoids, and polyphenol amides, among which anthocyanins and quercetin are flavonoids, and resveratrol is a nonflavonoid polyphenol found in grapes and red wine [120].

#### Anthocyanins

Anthocyanins are glycosylanthocyanidins that are widely distributed in plant vesicles, and their color depends on the pH of the environment [121]. A study reported that [122] anthocyanins can improve obesity, control diabetes, and prevent cardiovascular disease and cancer. Baoming Tian et al. [123]. reported that anthocyanins can increase the production of SCFAs by SCFA-producing bacteria, such as Ruminococcaceae, *Akkermansia*, *Bacteroides* and *Odoribacter*, and attenuate HFD-induced intestinal barrier damage by activating SCFA receptors, such as FFAR2 and FFAR3, and by upregulating TJ proteins. A study by Xu Si al [124]. revealed that blueberry anthocyanins attenuated the high-fat diet-induced oxidative stress state in mice. The levels of SCFAs in the intestine are promoted by increasing the SCFA-producing bacteria *Roseburia*, *Faecalibaculum*, and *Parabacteroides*, and increases in of SCFAs were shown to reduce hepatic steatosis and to improve the status of hippocampal neurons in mice. A study by Jiebiao Chen et al. [125]. showed that anthocyanins from common berries such as blackberries, black goji berries, strawberries, mulberries, prunes, raspberries, and red goji berries were able to increase the level of SCFAs in the intestine, improve the internal antioxidant status of mice, alleviate body weight gain and inhibit food intake by enriching the SCFA-producing bacteria *Ruminococcus*, *Intestinimonas*, and *Clostridium_XVIII*, among others. A study by Telma Angelina Faraldo Corrêa et al. [126]. showed that the intake of blood orange juice anthocyanins affected the abundance of operational taxonomic units (OTUs) in the intestinal flora, significantly increasing the levels of propionic acid and isobutyric acid in the intestinal tract of overweight females, reducing fasting blood glucose and insulin levels, and improving insulin resistance. A study by Ting Chen et al. [127]. found that purple red rice bran anthocyanins could promote the production of SCFAs in the intestinal tract and upregulate the expression of tight junction proteins (TJs) and nuclear factor kappa B (NF-κB) pathway proteins to improve the intestinal barrier function and dysbiosis of the intestinal flora in mice. A study by Yun Zhang et al. [128]. revealed that cactus anthocyanins significantly increased the content of SCFAs in the cecum of mice by altering the microbial diversity and flora composition of the intestinal tract, with the greatest increases observed in the contents of acetic acid, propionic acid and butyric acid.

#### Quercetin

Quercetin is a plant pigment that is widely found in tea, lettuce, radish leaves, cranberries, apples, buckwheat, cucumber, and onion and exists in plants as quercetin-3-O-glucoside, which provides color to a wide variety of vegetables and fruits [129, 130]. It has antiallergic, anti-inflammatory, cardiovascular protective, antitumor, antiviral, antidiabetic, immunomodulatory and antihypertensive effects [131]. Quercetin is also able to modulate metabolic disorders through different mechanisms, such as by increasing lipocalin, decreasing leptin, decreasing insulin resistance, increasing insulin levels, and blocking calcium channels [132]. A study by Wenhui Liu et al. [133]. reported that quercetin reduced the expression of transient receptor potential vanilloid 1 (TRPV1), aquaporin 3 (AQP3), and inducible nitric oxide synthase (iNOS) in the intestine and increased the expression of glial cell line-derived neurotrophic factor (GDNF), c-Kit, and stem cell factor (SCF). These changes inhibited the growth and reproduction of *Enterococcus* and *Enterobacter* in the intestine, increased the intestinal acetic acid, propionic acid and butyric acid contents in the intestine, improved gastrointestinal peristalsis, and increased the intestinal transit rate. Quercetin increases the abundance of *Coprococcus spp. Ruminiclostridium spp.* and *Roseburia spp.* in the mouse intestine [134] and the amount of these gut microbes that can produce SCFAs [135], which improves glucose and lipid metabolism in the body.

#### Resveratrol

Resveratrol was first isolated by Takaoka in 1939 from the flower Quercus serrata [136]. This phenolic substance is found not only in grapes and wine but also in peanuts, soybeans and berries [137]. It has powerful regenerative, antioxidant, protein-regulating and anticancer properties [138]. Early studies have shown that resveratrol inhibits the oxidation of low-density lipoprotein (LDL) in humans [139], reduces insulin resistance in animal models [140], and reduces adipocyte size and the inflammatory response in adipose tissue [141]. A study by Jin-Xian Liao et al. [142]. showed that resveratrol increased the abundance of *S24-7* and *Adlercreutzia*; decreased the abundance of *Allobaculum*, *Blautia*, *Lactobacillaceae*, and *Prevotella*; increased the concentration of acetic and propionic acids in the intestine; and regulated hepatic antioxidant capacity by increasing the expression of NF-E2-related factor 2 (Nrf2) in the liver to promote HO-1 transcription as well as the expression of superoxide dismutase (SOD) and Catalase (CAT). A study by Yu Zhuang et al. [143]. reported that resveratrol supplementation decreased expression of the inflammatory cytokines IL-6 and IL-1β; increased the expression of propionic acid, isobutyric acid, butyric acid and isovaleric acid in the intestinal tract; and affected the metabolism of the intestinal flora by regulating amino acid metabolism and lipid metabolism to improve intestinal health in mice. A study by Hongjia Yan et al. [144]. revealed that resveratrol ameliorated the progression of diabetic kidney disease by increasing the abundance of *Faecalibaculum* and *Lactobacillus* bacteria in the intestinal tract, increasing the concentration of acetic acid in the feces, and modulating the gut microbiota-SCFA axis. A study by Le-Feng Wanget al [145]. reported that resveratrol lowered the intestinal pH, promoted the growth and proliferation of probiotics in the intestinal tract, and significantly increased the content of isobutyric acid in the colons of aging mice.

### Carotenoids

Carotenoids are a general term for yellow, red and orange pigments containing long-chain hydrocarbons with conjugated double bonds. More than 1,100 carotenoids exist in nature, and humans can obtain them from vegetables and fruits [146]. Carotenoids can reduce free radical damage to cells [147] and are an important natural antioxidant. One molecule of beta-carotene can inhibit the activity of 1000 oxygen molecules [148]. Additionally, carotenoids can also be metabolized in the intestine and other tissues into vitamin A-like bioactive compounds, which provide immunomodulation and enhance the immune response [149]. Carotenoid supplementation activates the AMPK signaling pathway, which in turn activates upstream kinases, upregulates transcription factors, induces the discoloration of white adipose tissue, and blocks adipogenesis [150, 151]. Lycopene is a carotenoid [152] with antioxidant, anti-inflammatory, apoptotic and cellular communication-modulating effects [153]. A study by Xiang Gao et al. [154]. revealed that lycopene can decrease the expression of proteins such as NLRP3, Pro-Caspase-1, Caspase-1, and NF-κB in the liver; decrease the abundance of *Lachnospiraceae_NK4A136_group*, *Desulfovibrio*, and *Alistipes* in the gut microbiota; and increase the abundance of *Allobaculum* microbiota, thereby increasing the content of SCFAs, inhibiting the NF-κB/NLRP3 inflammatory pathway, and ameliorating nonalcoholic fatty liver disease (NAFLD). However, intake of beta carotene has been a question worth exploring, with studies concluding that 20 mg/day or more beta carotene is contraindicated for heavy smokers; the Panel on Nutrition, Dietetic Products, Novel Food and Allergy of the Norwegian Scientific Committee for Food Safety has set a tentative upper limit (TUL) of 4 mg/day for beta carotene supplementation; and the Panel on Vitamins and Minerals has set a safe upper limit of 7 mg/day for lifetime beta carotene supplementation for the general population (excluding smokers) and has discouraged concomitant use of beta carotene supplements by smokers [155].

### Betaine

Betaine is a stable and nontoxic natural substance that was first discovered in the plant *Beta vulgaris* and subsequently found in relatively high concentrations in several other organisms, such as wheat bran, wheat germ, spinach, and sugar beet [156]. Betaine [157] is a trimethyl derivative of the amino acid glycine that promotes glucose uptake through GLUT-4 expression, directly increases ATP production while helping to stimulate glucose utilization in myocytes, and enhances energy production by increasing mitochondrial biogenesis. Betaine can also play an important role as an antioxidant and protect the liver from oxidative stress [158]. A study by Jingjing Du et al. [159] revealed that betaine supplementation increases acetate- and butyrate-producing intestinal flora such as *A. muciniphila*, *Ruminococcus*, *Oscillospira*, and *Lactobacillus* in the gut, which in turn increases acetate and butyrate levels and improves the prevention of obesity and obesity-related metabolic comorbidities. A study by Liuqiao Sun et al. [160] revealed that betaine supplementation upregulated expression of hepatic lipid oxidation genes, such as PPARα and CPT1α, and the hepatic lipid transporter gene FATP2; downregulated expression of the adipogenic genes fatty acid synthase (Fas) and ACC in adipose tissue; decreased the relative abundance of *Proteus mirabilis*, *Vibrio desulfuricans*, and *Ruminococcus ruminanti* in the intestine; and increased the relative abundance of *Lactobacillus* and *Lactobacillus paracasei* in the intestine. Increasing the concentration of SCFAs in the feces can modulate the hepatic triglyceride content and improve NAFLD.

## Conclusions

Glucose and fatty acids are the main sources of energy in the human body. Under normal metabolic conditions, glucose metabolism and lipid metabolism pathways can meet the body’s normal activity needs, and the two metabolic pathways can affect each other; for example, glucose can be converted to fatty acids and cholesterol through the lipid biosynthesis pathway in glucose and lipid metabolism disorders, cardiovascular disease, diabetes, fatty liver and other serious diseases [161]. SCFAs modulate the structure of the gut microbiota [162], enhance the intestinal epithelial barrier [163], and can slow the onset and progression of disease [164]. By regulating the levels of SCFAs in the gut, it is able to exert beneficial effects on human glucose and lipid metabolism. Natural compounds in plants continue to be a hot topic in current research because of their safe composition, wide range of sources, and ability to treat a variety of diseases, such as cancer, diabetes, heart disease and Alzheimer’s disease [165]. Here, we review the role of SCFAs in glucose and lipid metabolism and describe the mechanism by which natural compounds in plants, such as polysaccharides, anthocyanins, quercetin, resveratrol, carotenoids, and betaines, modulate human glucose and lipid metabolism by increasing the content of SCFAs. Research has shown that these natural compounds can increase the number of beneficial bacteria, such as *Alistipes* and *Odoribacter*, thereby helping to maintain intestinal health. Moreover, they can also reduce potentially harmful bacteria, such as *Lactobacillus*, and lower their content in the intestine. Natural compounds can also regulate the content of short-chain fatty acids by affecting the abundance of OTUs in the gut microbiota and the intestinal transport rate. This regulatory effect helps to maintain the stability of the gut microbiota and prevent excessive proliferation of harmful bacteria. By using natural compounds, we can adjust the structure of the gut microbiota, maintain the stability of the intestinal environment, increase the content of SCFAs in the intestine, and promote human health. Further research on the effects of natural compounds on human SCFAs can help develop more beneficial products and methods for improving human health. Although the mechanisms by which natural compounds in plants regulate human glucose and lipid metabolism have been widely studied, the shortcomings of the results reported to date are that most were conducted in animal, with very few clinical trials. Moreover, the variety of natural compounds with SCFAs as unique targets of action is still limited. As a next step, preclinical and clinical studies are needed to understand and identify natural compounds that can play a role in regulating human glucose and lipid metabolism by modulating SCFAs. Additionally, targeted studies are needed to develop natural compounds in plants that provide new therapeutic options for the treatment of glucose and lipid metabolism disorders.

## Data Availability

No datasets were generated or analysed during the current study.

## Abbreviations

SCFAs: Short-chain fatty acid
SLC5A8: Anti-SLC5A8 rabbit polyclonal antibody
SLC16A1: Human solute carrier family 16, member 1
ATP: Adenosine triphosphate
GPR43: G protein coupled receptor 43
GPR41: G protein coupled receptor 41
G-6-pase: Glucose-6-phosphatase
SREBP1: Sterol regulatory element-binding protein 1
FGF21: Fibroblast growth factor 21
GLU-2: Glucose transporter 2
GYS2: Glycogen synthase 2
BAT: Brown adipose tissue
AMPK: Adenosine 5‘-monophosphate (AMP)-activated protein kinase
GLUT4: Glucose transporter 4
PFK-1: phosphofructokinase-1
GLP-1: Glucagon-like peptide-1
PYY: Peptide YY
PC1/3: Prohormone convertase 1/3
GCG: Recombinant Glucagon
HDAC: Histone deacetylase
ACC: Acetyl coenzyme A carboxylase
POMC: Proopiomelanocortin
NPY: Neuropeptide Y
AgRP: Agouti-related peptide
T2DM: Type 2 diabetes mellitus
IGN: Intestinal gluconeogenesis
cAMP: Cyclic Adenosine Monophosphate
G6PC: Glucose-6-phosphatase catalytic subunit
PCK1: Phosphoenolpyruvate carboxykinase 1
PGC1α: Proliferator–activated Receptor γ Coactivator 1α
PPARδ: Peroxisome Proliferator–activated Receptorδ
FAO: Fatty Acid 0xidation
mTOR: Mechanistic Target of Rapamycin
CTLs: Cytotoxic T lymphocytes
IFN-γ: Interferon Gamma
IL-2: Interleukin-2
TNF-α: Tumor Necrosis Factor Alpha
Slc2a1: Glucose Transporter 1
PPARα: Peroxisome proliferators-activated receptors Alpha
ChREBP: Carbohydrate response element binding protein
HSL: Hormone-sensitive triglyceride lipase
UCP-1: Uncoupling protein 1
PPARγ: Peroxisome Proliferator-activated Receptorγ
UCP-2: Uncoupling protein 2
UCP-3: Uncoupling protein 3
CPT1-b: Carnitine Palmitoyltransferase 1b
IL-15: Interleukin-15
IL-1β: Interleukin-1β
IL-6: Interleukin-6
TNF-α: Tumor necrosis factor alpha
OTU: Operational Taxonomic Unit
HFD: High-fat diet
TJs: Tight junction proteins
NF-κB: Nuclear factor kappa B
TRPV1: Transient Receptor Potential Vanilloid 1
AQP3: Aquaporin 3
iNos: inducible Nitric Oxide Synthase
GDNF: Glial Cell Line-derived Neurotrophic Factor
SCF: Stem Cell Factor
Nrf2: NF-E2-related Factor 2
SOD: Superoxide Dismutase
CAT: Catalase
CPT1ɑ: Carnitine lipoyltransferase Iα
FATP2: Fatty acid transport protein 2
